# Perspectives on Endosymbiosis in Coralloid Roots: Association of Cycads and Cyanobacteria

**DOI:** 10.3389/fmicb.2019.01888

**Published:** 2019-08-14

**Authors:** Aimee Caye G. Chang, Tao Chen, Nan Li, Jun Duan

**Affiliations:** ^1^University of Chinese Academy of Sciences, Beijing, China; ^2^Fairy Lake Botanical Garden, Chinese Academy of Sciences, Shenzhen, China; ^3^South China Botanical Garden, Chinese Academy of Sciences, Guangzhou, China

**Keywords:** cyanobacteria, cycads, coralloid roots, symbiosis, cyanobionts, endosymbiosis, heterocyst, hormogonia

## Abstract

Past endosymbiotic events allowed photosynthetic organisms to flourish and evolve in terrestrial areas. The precursor of chloroplasts was an ancient photosynthetic cyanobacterium. Presently, cyanobacteria are still capable of establishing successful symbioses in a wide range of hosts. One particular host plant among the gymnosperms is cycads (Order Cycadales) in which a special type of root system, referred to as coralloid roots, develops to house symbiotic cyanobacteria. A number of studies have explained coralloid root formation and cyanobiont invasion but the questions on mechanisms of this host-microbe association remains vague. Most researches focus on diversity of symbionts in coralloid roots but equally important is to explore the underlying mechanisms of cycads-*Nostoc* symbiosis as well. Besides providing an overview of relevant areas presently known about this association and citing putative genes involved in cycad-cyanobacteria symbioses, this paper aims to identify the limitations that hamper attempts to get to the root of the matter and suggests future research directions that may prove useful.

## Introduction

Cyanobacteria are the ancestors of chloroplasts. Gaining a deeper understanding on how communication between a symbiont and a host occurs at the molecular level may provide insights on the evolution of green plants. Studies addressing the function and symbiotic mechanisms between cycads and cyanobacteria in coralloid roots are scant when compared to studies of associations of cyanobionts with other host plants such as the angiosperm *Gunnera*, the water fern *Azolla* and the bryophytes *Blasia* and *Anthoceros*, to cite a few (Adams et al., 2013; Warshan, 2017). Most of the literature on cyanobacterial associations with cycads are old. Recent publications (Bergman et al., 1992; Kluge et al., 1992; Adams and Duggan, 2008; Rikkinen, 2015; Pereira, 2017) based their analyses on other host-symbiont models in an attempt to explain this ancient partnership. Therefore, this review aims to summarize current knowledge about endosymbiosis and highlighting past and present work on cycads-cyanobacterial associations. A section is also dedicated to briefly citing symbiosis-associated genes already identified from other symbiotic models that are relevant in trying to determine the core functions and mechanisms of maintaining stable symbiotic relationships. This review targets readers whose knowledge about coralloid roots of cycads are limited with the aim to spark interest in this infrequently-studied root symbiosis. This paper also aims to tackle the hindrances in this field of research and to suggest where future research may prove productive.

### Early Symbiosis

The capacity of various microbes to interact with other life forms existed even before plants flourished – an evolutionary event triggered by symbiotic microbes (Suárez-Moo et al., 2019). Plastids originated from cyanobacteria (Douglas, 1998), also known as blue green algae, through the process of endosymbiosis which gave rise to the photosynthetic eukaryotes we now refer to as plants and algae (de Vries and Gould, 2017). Chloroplasts became an important component of plant cells because during the past endosymbiotic event by which a heterotrophic unicellular protist engulfed a free-living photosynthetic organism, the former did not allow digestion of the latter – a cyanobacterium. This is the brief story of how plastids originate (Chan and Bhattacharya, 2010). The endosymbiotic cyanobacterium became a plant organelle that harnesses solar energy and converts it to sugar and starch for food and in the process generated the oxygen in the atmosphere that is vital to most life forms (Sarma et al., 2016). In contrast to how free-living cyanobacteria evolved to become chloroplasts (de Vries and Gould, 2017), the event of symbiotic cyanobacteria that entered the roots of cycads had a different outcome. Instead of co-evolving and the symbiont being inherited intracellularly by the host from generation to generation, or giving rise to another organism, symbiotic cyanobacteria just enter and reside in cycad roots in a later part of its host’s development (Norstog and Nicholls, 1997) in an engorged and dichotomously-branched root modification called coralloid roots (Ahern and Staff, 1994). Similar to this is the evolution of modified plant roots observed in pines (Faye et al., 1981) and legumes (Long, 1989) to house their respective microsymbionts for a specialized function (Suárez-Moo et al., 2019). Still, regardless of how symbionts invade and evolve in their hosts, life as we know it would not have flourished if not for endosymbiosis (Govindjee and Shevela, 2011; Shestakov and Karbysheva, 2017). Research focusing on endosymbiosis is interesting, since intimate associations between two unrelated species may trigger another critical event that might cause changes in the Earth’s atmospheric composition which would impact many life forms (Raven and Allen, 2003; Green, 2011; Martin et al., 2015).

Based on evidence obtained from fossils, cyanobacteria are known to be among the earliest groups of microorganisms that dominated the Earth since the late Archean to early Paleoproterozoic eon around 3500 to 2700 MYA (Krings et al., 2009; Falcon et al., 2010; Schopf, 2011; Shestakov and Karbysheva, 2017). Free-living and symbiotic cyanobacteria vary from spherical and cylindrical unicellular to filamentous multicellular forms (Narainsamy et al., 2013). Known symbiotic cyanobacteria are mostly filamentous members of the genus *Nostoc* (Adams and Duggan, 2008).

Cycads are the only members of gymnosperms currently capable of forming new associations with cyanobacteria. Initially reported by Reinke in 1872 (cited in Adams et al., 2013), all known species of cycads form symbiotic associations with cyanobacteria in specialized structures called coralloid roots. Similar to cyanobionts (endosymbiotic cyanobacteria), cycads belong to the earliest members of the five major groups of seed plants – the gymnosperms Cycadales, Coniferales, Ginkgoales, Gnetales, and angiosperms. Cycads are sisters with *Ginkgo* (Wu et al., 2013) and both share a common ancestor with gnetophytes and conifers (Wu et al., 2007; Roodt et al., 2017). Cycads are assumed to have coexisted with dinosaurs during the Mesozoic era around 300 MYA and thus are often referred to as “living fossils” (Wu et al., 2007; Wu and Chaw, 2015; Jiang et al., 2016). Since cycads and cyanobacteria date back to ancient times, the symbiotic partnership and coevolution formed between the two may have developed millions of years ago (Usher et al., 2007). The origin of this association is still debated and questions regarding the purpose of this symbiosis and why it still prevails remains to be answered.

### Implications of Symbiosis Between Cycads and Cyanobacteria

Since ancient soils 300 million years ago (Brenner et al., 2003) were not as fertile as they are today, it was thought that cycads developed a mechanism to harbor cyanobacteria to withstand poor-nutrient soils (Halliday and Pate, 1976). Specialized root structures to house endosymbiotic cyanobacteria were formed and a mutualistic association was maintained by both partners (Gutiérrez-García et al., 2019). Whether coralloid roots were formed by cycads only for the purpose of hosting cyanobionts is an open question. As opposed to cycads, terrestrial cyanobacteria can live in diverse and harsh environments (Sand-Jensen, 2014) and are considered to be the most successful group of microorganisms on Earth (Stewart and Falconer, 2011). Free-living cyanobacteria also form associations with other life forms such as plants and fungi and in some cases with tripartite-structured cyanolichens made up of fungi, green algae and cyanobacteria (Henskens et al., 2012). As to why they need a host to spend a part of their life cycle, some scientists believe that terrestrial cyanobacteria also prefer a stable environment to survive and prevent predation and desiccation from intense heat (Adams et al., 2013). Thus, mutualistic relationships between hosts and endosymbionts are formed wherein the host provides shelter while the symbiont performs specialized functions, such as supplying the host with various needs (Walsh et al., 2011; Haselkorn, 2016). Additionally, it was suggested that cyanobacteria produce arabinogalactan-proteins (AGPs) that contribute to helping plants grow and develop (Pennell, 1992) by assisting in plant cell proliferation, expansion and differentiation (Steele-King et al., 2000). Moreover, AGPs are known to have a role in cell signaling in plant-microbe interactions (Seifert and Roberts, 2007; Jackson et al., 2012).

In a symbiotic relationship, cyanobacteria fixes nitrogen for their hosts. Naturally-occurring dinitrogen in the atmosphere is unreactive with other chemicals, thereby preventing formation of essential and useful compounds. This is where endosymbiotic cyanobacteria enter the Earth’s nitrogen cycle and play a major role. Cyanobionts are able to break down the triple bonds from atmospheric dinitrogen (N_2_) using the enzyme nitrogenase to convert the inert compound into useful forms of nitrogen. The N_2_ atoms can then be converted to ammonia (NH_3_) (Hoffman et al., 2014), which facilitates plant growth and soil fertilization (Halliday and Pate, 1976). Other nitrifying microbes such as *Nitrospira* sp. assist in oxidation of ammonia to nitrite and nitrate followed by the action of denitrifying microbes that completes the nitrogen cycle (Kuypers, 2015). Nitrogen fixation in *Nostoc*, the dominant species symbiotic to cycads coralloid roots (Gehringer et al., 2010), occurs in structures called heterocysts, which occur as chain of cells forming a filament. Sufficient evidence shows that nitrogenase activity of cyanobacteria in symbiosis is significantly higher than in their free-living counterparts, as shown by a 25–35% increase in heterocyst formation in cyanobionts (Lindblad et al., 1985a).

**FIGURE 1:**
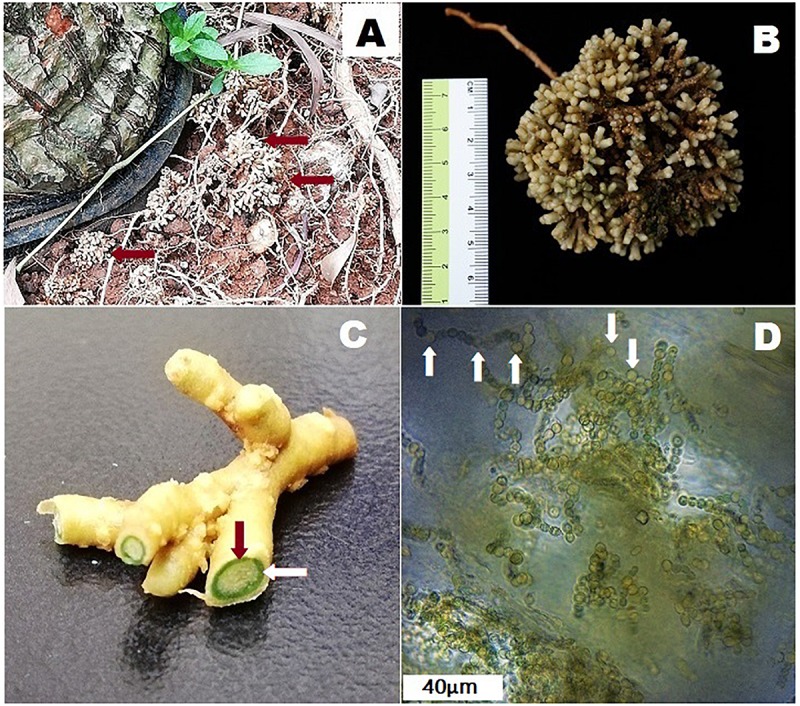
Coralloid roots of *Cycas fairylakea* **(A)** growing apogeotropically at Shenzhen Fairy Lake Botanical Garden, **(B)** showing dichotomous branching of coralloid roots, **(C)** cross-section of coralloid root showing a distinct green layer – the cyanobacterial zone (the green ring pointed by the red and while arrows) and **(D)** microscopic image of filamentous cyanobacteria residing in the cyanobacterial zone. Heterocysts are marked with white arrows.

Studies show that cyanobionts in symbiosis with cycads maintain complete photosynthetic apparatus – thylakoids, phycobilisomes, phycobiliproteins, and carboxysomes, associated pigments and enzyme levels comparable with free-living cyanobacteria (Lindblad et al., 1985b; Adams et al., 2013). However, as coralloid roots grow beneath the soil surface where light is insufficient or lacking, the photosystems of cyanobionts may be inactive as shown in an *in vivo* study conducted by Lindblad et al. (1987) where coralloid roots of *Cycas revoluta* showed no evidence of carbon fixation activities when compared under light and dark conditions. It was suggested that enzymes specific for efficient function of the Calvin cycle may be missing in the cyanobacteria in coralloid roots (Lindblad et al., 1987). Since cyanobionts have no sufficient light source, they are capable of heterotrophic metabolism relying on carbon solely supplied by their hosts (Lindblad, 2009). However, it is noteworthy that coralloid roots grow apogeotropically as if phototropism is occurring, and based on personal observations (Figure 1A), they oftentimes reach the soil surface exposed and thus, may become capable of receiving substantial amount of light probably through dermal breaks.

The association of cyanobacteria and cycads was also found prevalent in an incident in Guam that led to the detection of neurotoxins contained in cycad seeds. The neurotoxins cause an amyotrophic lateral sclerosis/parkinsonism-dementia complex (ALS-PDC) (Brownson et al., 2002; Meneely et al., 2016). ALS-PDC is a progressive neurodegenerative disease that infected high number of Chamorro people in Guam (Metcalf et al., 2017; Zurita et al., 2019). The disease was believed to be transmitted by flying foxes that consumed cycad seeds. Flying foxes are eaten by the inhabitants of the island (Cox and Sacks, 2002; Banack and Cox, 2003). A case of “biomagnification” was said to have occurred wherein the toxin becomes more potent after being transferred from one organism to the other (Banack and Cox, 2003; Cox et al., 2003; Banack et al., 2006). But this premise remains highly debated (Snyder and Marler, 2011). In contrast, Marler et al. (2010) suggested that coralloid roots do not synthesize or biomagnify these toxins but rather, may act as toxin sinks explaining the high amounts of toxins found in the coralloid root tissues obtained from previous studies. Cyanobacteria-free cycad seedlings were in fact, found to increase in these toxins compared to symbiont-infected coralloid roots therefore refuting the role of endophytic cyanobacteria causing increase of these toxic substances in the host plant (Marler et al., 2010; Snyder and Marler, 2011). Free-living cyanobacteria are known to produce various toxins and thus it is not surprising that symbiotic cyanobacteria synthesize toxins as well (Cox et al., 2005). This neurotoxin was claimed to be β-methylamino-L-alanine (BMAA), a non-proteinogenic amino acid produced by cyanobacteria. Even though some studies have showed that cycad host plants contain BMAA (Vega and Bell, 1967; Polsky et al., 1972; Brownson et al., 2002; Banack and Cox, 2003; Cox et al., 2003), an accurate detection method of BMAA is still being perfected until the present (Metcalf et al., 2017; Zurita et al., 2019) due to various structural isomers (Rosén and Hellenäs, 2008; Banack et al., 2010; Jiang et al., 2012) making it difficult to correctly identify the compound as BMAA. And thus, all previous claims that BMAA was detected still needed further validation.

### Cyanobacterial Diversity and Development in Cycads

Cyanobionts found in cycads are predominantly species of *Nostoc*, but in some studies, species of *Calothrix*, *Scytonema* and *Richelia* were also identified (Grobbelaar et al., 1987; Costa and Lindblad, 2002; Gehringer et al., 2010). Most symbiotic cyanobacteria belong to the orders Nostocales and Stigonematales (Castenholz et al., 2001). Multiple strains of cyanobacteria can be housed in a single cycad host (Zheng et al., 2002; Thajuddin et al., 2010) and a single species of cyanobacterium can be isolated in multiple cycad hosts as well (Gehringer et al., 2010). However, in a study by Gehringer et al. (2010), only a single symbiotic *Nostoc* strain was found harboring the coralloid roots of the genus *Macrozamia* (Yamada et al., 2012). Reports indicate that other heterotrophic bacteria reside with cyanobacteria in coralloid roots (Chang et al., 1988), but only in limited populations. According to Grilli Caiola (1980), this is due to the ability of cycads to produce secondary metabolites that inhibit the growth of microorganisms, but not cyanobacteria. A concerted communication between the host and a bacterium, probably through production of certain substances, may also play a role in preventing other bacteria to overpopulate the cyanobacterial layer inside the coralloid roots (Obukowicz et al., 1981; Adams et al., 2013).

Cycads form three types of roots: (1) a primary tap root similar to the root system of most terrestrial plants, (2) lateral roots and (3) coralloid roots (Figure 1B). The latter are distinct types of roots that grow laterally and are solely in cycads that house cyanobacteria (Norstog and Nicholls, 1997; Lindblad, 2009). Prior to coralloid root formation, young, apogeotropic papillose roots called precoralloid roots are formed (Ahern and Staff, 1994). During this phase, cyanobacteria are absent and their presence is not required in initiating the development of precoralloids. At this early stage, invasion by cyanobionts happen but note that cases of uninfected precoralloids also occur (Milindasuta, 1975). This raises the question whether precoralloid roots are formed by cycads to specifically host cyanobacteria or also to serve another purpose. Nevertheless, the affinity of cyanobacteria to enter into a symbiotic relationship with coralloid roots instead of with the primary and lateral roots promotes the idea that precoralloid and coralloid roots are organs developed by cycads to facilitate symbiosis. The tips of cyanobacteria-free precoralloid roots produce papillose tissue continuously (Ahern and Staff, 1994). Upon maturity, the sheath covering the papillose tissues will be replaced by a thin, external layer that generates scattered lenticels (Milindasuta, 1975; Ahern and Staff, 1994). These morphological changes, or other environmental factors, may cause disruptions in the dermal tissues of mature precoralloid roots. When cyanobacteria in the surrounding soil come in contact with the surface of coralloid roots, they gain access through the dermal breaks (Milindasuta, 1975) to eventually colonize the internal layers of the roots (Grilli Caiola and Canini, 1993; Ahern and Staff, 1994; Lindblad, 2009). At this point, the morphologically distinguishable, engorged and dichotomously-branching coralloid roots start to form. Following initial entry, cyanobacteria migrate toward the cortex and form a distinct, circular, blue-green layer dividing the cortical layer into two (Ahern and Staff, 1994). This is called the cyanobacterial zone (Figure 1C) containing filamentous cyanobionts (Figure 1D). When coralloid roots reached this stage, the process is irreversible and a permanent symbiotic relationship between cycads and cyanobacteria has been successfully established (Grilli Caiola and Canini, 1993; Ahern and Staff, 1994; Lindblad, 2009).

### Associated Structures Required for Establishment of Symbiosis

Cyanobacteria that can form associations with cycads as well as with other compatible host plants are capable of cell differentiation exhibiting various morphologies (Flores, 2012). All symbiotic cyanobacterial strains from the genus *Nostoc* fix nitrogen in cells called heterocysts (Adams and Duggan, 2008). At low levels of nitrogen, cyanobionts form heterocysts to facilitate nitrogen metabolism (Bergman et al., 1996). Forming heterocysts was how nitrogen fixers evolved to protect the enzyme nitrogenase from inactivation due to exposure to oxygen (Bernhard, 2010). These cells are thick-walled allowing them to block available oxygen in diffusing inside the cells making a suitable, low-oxygen microenvironment for efficient synthesis of nitrogenase for nitrogen metabolism (Tikhonovich and Provorov, 2007). Heterocysts are specialized elliptical-shaped cells produced at regularly-spaced intervals along the cyanobacterial linear cell clusters and are distinguishable due to their larger size compared to neighboring vegetative cells (Kumar et al., 2010). Cyanobionts in coralloid roots form heterocysts at higher frequency compared to free-living cyanobacteria but morphological changes are minimal (Adams et al., 2013). For cyanobacteria living within coralloid roots, a significant increase (up to 80%) in heterocyst frequency was observed (Norstog and Nicholls, 1997; Adams and Duggan, 1999; Zhang et al., 2006). The increase in formation of heterocysts is said to be triggered by nitrogen starvation (Zhang et al., 2006). Therefore, an effective partnership with a plant host relies on the ability of the cyanobacteria to differentiate into heterocysts for a stable symbiosis (Meeks and Elhai, 2002).

Cyanobacteria can also form filaments called hormogonia. Hormogonium lacks heterocysts and can be morphologically distinguished from the latter as the former is capable of locomotion appearing as short motile filaments (Hernández-Muñiz and Stevens, 1987). Hormogonia play a role in self-dispersal and in forming symbiotic association during the early stages of infection by responding to chemical signals produced by prospective host plants (Khayatan et al., 2017). Chemoattractants are said to be involved in stimulating hormogonia formation and directing the cyanobacteria toward the targeted plant tissue that will house the cyanobionts (Meeks et al., 2001; Meeks and Elhai, 2002; Bergman et al., 2007).

Filamentous, heterocyst-forming cyanobacteria are also capable of differentiating into spores called akinetes (Kaplan-Levy et al., 2010; Adams et al., 2013). When environmental conditions become unfavorable (e.g., low salinity, temperature fluctuations, lack of phosphates, low light, insufficient nutrients), active cells transform into a resting state that may last for up to 60 years in which they can be restored to their vegetative cell state when favorable conditions arise (Kaplan-Levy et al., 2010). Although not resistant to intense heat, akinetes can survive cold and desiccation (Adams and Duggan, 1999). Akinetes are rare but were reported to occur in strains of *Nostoc* isolated from coralloid roots (Grilli Caiola, 1980; Grobbelaar et al., 1987) and more are common in *Azolla-Anabaena* symbioses (Peters and Perkins, 1993). Additionally, Sukenik et al. (2007) state that resting cells can still perform minimal metabolic activities such as photosynthesis, carbon fixation and protein synthesis. Aside from akinetes, lysing or dying cells called necridia, which allow excision of cells along the filament, also occur in cyanobacteria. Characterized by thickened walls and non-granulated cells, these are commonly referred to as deteriorating cells that were unable to differentiate into heterocysts (Grilli Caiola and Pellegrini, 1979).

### General Mechanism of Symbiosis

**FIGURE 2:**
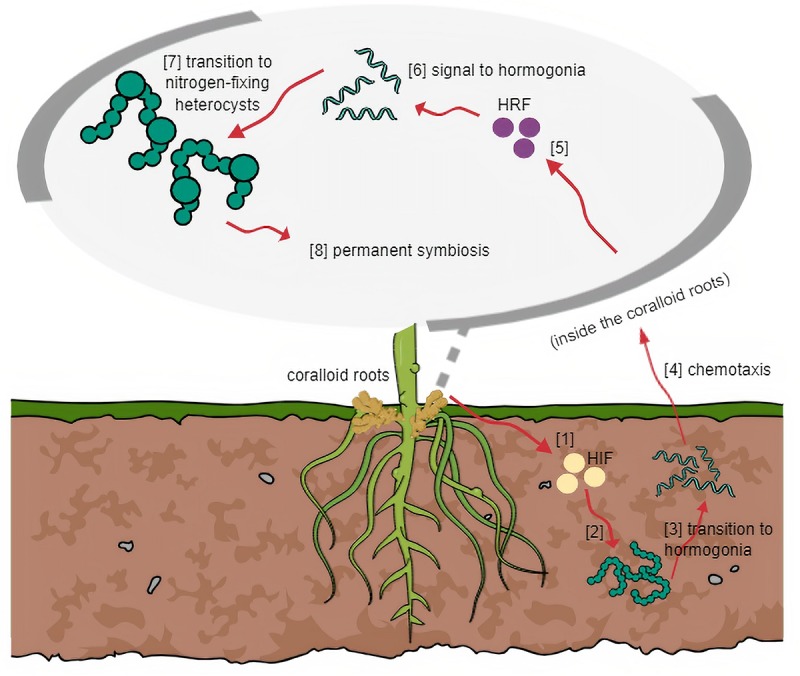
General mechanism of symbiosis based on other host plant-cyanobiont models. (1) HIF is released by host and (2) triggers cyanobacteria in surrounding soil to (3) transition into motile filaments. (4) Through chemotaxis, host attracts hormogonia following cyanobacterial entry. (5) Host releases HRF and (6) signals hormogonia that entered the host successfully to (7) transition into heterocysts-forming cells. (8) Symbiosis at this stage becomes irreversible.

All known plant-cyanobacterial symbioses are acquired from the environment. The only exception is seen in the fern *Azolla*, where the cyanobiont is an integral part of the host throughout its developmental stages and gets inherited to the next generation (Bergman et al., 1996). Thus, for most symbioses, efficient communication between the host and cyanobacterium must be carried out to ensure successful entry of the symbiont (Figure 2). This requires signal molecules induced by the host and/or the symbiont during the initial stages of invasion. The general mechanism based on other host plant-cyanobacterial symbioses, as controlled by a set of regulatory genes, is that the host elicits hormogonium-inducing factors causing the surrounding cyanobacteria to transform into motile hormogonia (Adams and Duggan, 2012; Warshan, 2017). Chemotactic signals permit entry into the partner plant, subsequently demanding the host to produce hormogonium-repressing factors to allow the cyanobacteria to develop heterocysts for nitrogen fixation to occur (Warshan, 2017).

Formation of motile hormogonia is essential for successful migration of a cyanobiont to the internal tissues of its potential plant partner. Certain compounds which may be produced by the host, trigger this phenomenon and initiate cell differentiation (Khayatan et al., 2017). In plants known to accommodate symbiotic cyanobacteria, modified structures can be formed regardless of whether a cyanobiont will be present in its life cycle (Meeks and Elhai, 2002) such as the case in coralloid roots. Unlike *Rhizobia*, which requires specialized symbiosomes to be formed by its host, cyanobionts of coralloid roots do not need similar structures to survive within its host. However, particular spaces are dedicated to symbionts. Cyanobacteria resides in the cyanobacterial zone in the roots of cycads, in the red stem glands of *Gunnera*, in dorsal leaf cavities of *Azolla* water fern, in slime cavities within the thallus of hornworts, in auricles underneath the thallus of liverworts, and in the bladders of the fungi *Geosiphon pyriformis* (Adams et al., 2013). Among these plant-cyanobacterial symbioses, it is only in *Gunnera* where the cyanobiont invasion occurs intracellularly (Meeks and Elhai, 2002; Adams et al., 2013). In the coralloid roots, an acidic viscous mucilage in the cortex (Grilli Caiola, 1980), also observed by the authors, is present in the cortex layer probably involved in attracting motile hormogonia filaments as this was the case observed in *Gunnera* (Nilsson et al., 2006). Mucilage-filled cavities were also observed in hornworts and liverworts and this heat-labile putative signal inducer is yet to be characterized, but was estimated to be a low molecular mass protein around 12 kDa (Adams et al., 2013). In line with this, root extracts from cycad coralloid roots were found to significantly initiate hormogonia formation in strains of *Nostoc* (Campbell and Meeks, 1989; Bergman et al., 1996; Meeks, 1998) suggesting hosts produce hormogonia-inducing factors (Nilsson et al., 2006). In an *in vitro* study, hormogonia-inducing factor (HIF) was not released in a culture medium with excess nitrogen. Thus, HIF production is assumed to be stimulated when nitrogen levels are depleted (Campbell and Meeks, 1989; Adams et al., 2013). Aside from the HIF signal, plant hosts are believed to release various chemoattractants to entice potential symbionts. These were hypothesized to be sugar-based molecules, since simple sugars were proven to be attractants for hormogonia initiation (Nilsson et al., 2006; Adams et al., 2013). Recently, a bioassay was conducted using crude methanolic extracts of *Cycas revoluta* coralloid roots that transformed symbiotic *Nostoc* filaments into motile hormogonia. This led to the successful isolation of a hormogonium-inducing factor from cycads characterized as diacylglycerol 1-palmitoyl-2-linoleoyl-*sn*-glycerol (Hashidoko et al., 2019).

Once a cyanobiont successfully enters its host, the plant partner must stop releasing hormogonia-inducing factors to halt hormogonia formation. The reason is to direct the cyanobiont to the next stage of symbiosis and to start forming heterocysts for nitrogen fixation to occur – a task that cannot be performed by hormogonia filaments. Therefore, the release of hormogonia-repressing factors (HRF) hinders HIF activities. Cohen and Meeks (1997) observed this phenomenon using aqueous tissue extracts of *Anthoceros* wherein activation of two genes blocks hormogonium formation and the expressions are only induced by HRF but not HIF.

Besides inducing and repressing factors, high amounts of phenolic substances are also present in the mucilaginous material embedded in the cyanobacterial zone of coralloid roots and surrounding cortical layers (Lobakova et al., 2004). Phenolic substances are rich sources of antimicrobial compounds and secondary metabolites (Obukowicz et al., 1981). Interestingly, phenolics are also known to participate in cell signaling and might play a role in establishing and maintaining stable cycad-cyanobacterial symbioses (Grilli Caiola, 1980; Obukowicz et al., 1981).

### An Overview of Genes Involved in Cycads-Cyanobacteria Symbiosis

Though still limited, sequencing of representative complete genomes of hosts and cyanobionts led to the identification of genes involved in plant host-cyanobacterial symbiosis (Ran et al., 2010; Li et al., 2018). This section aims to list some studied genes and their functions significant in the establishment of symbiosis as well as other genes they interact with (Table 1). Although most of the genes cited here were not studied using cycad coralloid roots, a similar, if not exact, genetic mechanism in cycad-cyanobacterial symbiosis seems to be occurring as the genes discussed in this section were commonly found among plant-cyanobacterial symbioses.

**TABLE 1:** Symbiosis-related genes from host-symbiont models cited in this manuscript.

**GENES**	**ORGANISMS/s**	**GENERAL FUNCTION/S**
*hrm*	*Nostoc-Anthoceros*	Suppresses hormogonia formation allowing cell differentiation into heterocysts
*het*	*Nostoc-Anthoceros*	Responsible for heterocyst formation
*ntc*	*Nostoc-Anthoceros*	Regulates nitrogen and has a role in activation of other symbiotic genes
*hep*	*Anabaena-Azolla*	Induces formation of thickened cell envelope in heterocyst cells
*nif*	*Nostoc-Anthoceros, Anabaena-Azolla*	Involves in formation of nitrogenase complexes
*sig*	*Nostoc-Anthoceros*	Plays a role in signaling to promote symbiont entry to host tissue
*ctp*	*Nostoc-Anthoceros, Synechocystis-Anthoceros*	Involves in photosynthetic activities
*tpr*	*Nostoc-Anthoceros*	Regulates cell cycle and functions in protein transport mechanisms

As mentioned previously, HRF are involved in halting hormogonium formation after initial migration of the symbiont into its target organ in the host that varies among plant hosts as mentioned in the previous section. Using *Anthoceros* tissue extracts, *hrmU* and *hrmA* genes were discovered that suppresses hormogonia formation and allow differentiation of cells into heterocysts (Cohen and Meeks, 1997). The expression of these two genes appears to be controlled by the host, which hinders further activities attempted by HIF (Cohen and Meeks, 1997; Meeks et al., 1999). Other open reading frames (ORFs) – *hrmI, hrmR, hrmK* and *hrmE* – were identified that were similar to a family coding for transcriptional inhibitor proteins (Campbell et al., 2003). It was proposed that external repressing signals can possibly be detected by *Nostoc* incapacitating Hrm proteins in successfully binding to hrm operons and this affects transcription of genes that induce hormogonium formation (Meeks et al., 1999; Campbell et al., 2003; Adams et al., 2013).

The ability to form heterocysts is a vital feature for a cyanobiont to establish symbiosis with a host. Without heterocysts, a cyanobiont cannot fix nitrogen for its host. Mutations in *hetR* and *hetF* did not allow one strain of *Nostoc* to differentiate into heterocysts and thus, those genes were determined to be directly responsible for heterocyst formation (Wong and Meeks, 2001). The main activator for the development of heterocysts is the *hetR* gene, which works simultaneously with *hetF* gene coding, which gives rise to a protein that enhances subsequent transcription of *hetR* (Wolk, 2000; Wong and Meeks, 2002; Zhang et al., 2006). The expression of *ntcA* gene is necessary for the production of proteins that cyanobacteria use to regulate nitrogen and in addition, is involved in activating the transcription of various genes, including the *hetR* gene (Herrero et al., 2001). Studies showed that a mutant species of *Nostoc* with a defective *ntcA* gene failed to infect its host despite retention of hormogonia-forming capacity and therefore might be linked with the activation of other genes required for a successful symbiotic relationship to be established (Vega-Palas et al., 1992; Herrero et al., 2001; Adams et al., 2013). A study by Leganes et al. (1994) in *Anabaena* showed that an altered *hepA* gene disrupts proper formation of the cell envelope in both heterocysts and akinetes. The thickened walls, made up of a polysaccharide layer, are essential for nitrogen fixation activities to be concentrated in the heterocyst cells and prevent oxygen diffusion (Tikhonovich and Provorov, 2007). As a consequence of a non-functional *hepA* gene, normal development of the cell envelope is impossible and affects the ability of the cyanobiont to fix nitrogen in its host (Leganes et al., 1994). Both free-living and symbiotic bacteria have *nif* genes not restricted to cyanobacteria (Corbin et al., 1982; Fay, 1992). Those genes are responsible for forming nitrogenase complexes that convert unusable atmospheric dinitrogen to useful forms like ammonia and in this process, the *nifD* and *nifK* genes encode a dinitrogenase heterotetramer that contains an active site to reduce dinitrogen atoms (Dos Santos et al., 2012). The *nifDK* genes work together with it redox partner, an iron protein encoded by *nifH* gene, to complete the structure and function of nitrogenase complex (Jasniewski et al., 2018). Therefore, these genes are important for maintaining mutual associations with a host because defects in the genes may affect the nitrogen-fixing capacity of the symbiont (Spaink, 1998).

Several genes have been identified, but their significance in symbioses is still poorly understood. A sigma factor gene, *sigH*, which appears to be directed by HIF, might play a role in the ability of symbionts to increase invasion success in a targeted host (Campbell et al., 1998). Likewise, the *ctpH* gene, which is in close proximity with the *sigH* gene, is also controlled by HIF in *Nostoc* (Adams et al., 2013) and has a function in the photosystem II mechanisms in *Synechocystis* (Anbudurai et al., 1994). This gene is interesting as it may have varying physiological roles in different symbiotic strains. Another gene, *tprN*, codes for proteins known to be necessary in various functions, such as regulating the cell cycle, suppressing transcription mechanisms and transporting proteins (Lamb et al., 1995). This gene is also essential for heterocyst maturation process (Campbell et al., 1996). In line with this, Meeks et al. (1999) observed that a silenced *tprN* gene in *N. punctiforme* caused no phenotypic change but the infection rate doubled compared to the wild-type strain, and its transcription elevated when exposed to both HIF and HRF exudates of *A. punctatus*. However, the implications and its involvement in the infection process are still unknown.

## Limitations in Cycad-Cyanobacteria Research

A substantial number of studies have focused on the growth and development of coralloid roots and the diversity of the endosymbiont community within the cyanobacterial zone using cultures and 16S identification methods (Costa et al., 1999; Thajuddin et al., 2010; Yamada et al., 2012). The underlying mechanisms, however, have been left unexplored. Probable reasons challenging the study of symbiosis compared to other hosts with a cyanobacterial partner are discussed in this section.

One of the difficulties in this field of research is studying the *in vivo* development of cycad – cyanobacterial symbiosis. To determine the mutualistic interactions exhibited by both partners, a significant length of time, which may take years of research, might be needed to confirm and validate hypotheses as development in cycads takes much time. Tissue culture methods are also lacking for most cycads to facilitate *in vitro* experimental assays that could be used to compare wild-type and symbiont-free conditions. Another reason is the availability of coralloid roots of good quantity and quality. According to McLuckie (1922) cited in Milindasuta (1975) and also based on personal observations, even when coralloid roots are present, a green layer in the cortex may be absent (Nilsson et al., 2006). Moreover, the availability of both root tissue samples and cyanobionts per plant host are often insufficient for experiments, especially when replicates are needed. Thus, samples are usually pooled using a number of representative hosts. Also, most studies obtain their samples from botanic gardens and not from the wild. Although promising results can still be achieved, gathering samples from natural habitats could add more interesting findings.

Another frustration in this field of research is trying to separate root tissues from the endosymbiont as well as the endosymbiont from the sticky mucilaginous material. Various methods have been proposed (Ferris and Hirsch, 1991; Lindblad et al., 1991; Gehringer et al., 2010), but completely separating them from each requires improvement as most requires manually scraping off the green symbiont from the roots. In determining the functions of cycads and their cyanobionts during symbiosis, the gene expression must be analyzed capturing the symbiotic condition while both partners are together. This eliminates the need to separate them because it would cause changes in gene expression, but poses the more difficult task of how to monitor their association *in vivo*.

## Conclusion and Future Perspectives

Research on coralloid roots is not new, but progress has been quite slow compared to research on *Rhizobia* and other host-cyanobiont associations. Due to the limitations mentioned above, the research field has been confined to studying the diversity of symbionts obtained from various cycad hosts and revalidating previous hypotheses on how both partners benefit from each other. Cruz-Morales et al. (2017) identified novel biosynthetic gene clusters unique to cycad coralloid roots-cyanobacteria symbiosis through genome mining, a research area worth undertaking to understand coevolution and to discover pathways responsible for the synthesis of natural products. With the advancements in genomic research that are becoming more affordable, even for small-scale laboratories, studying cycad host-cyanobiont symbiosis may now move forward at a quicker pace. First, whole genome and transcriptomic sequencing proved to be a valuable source of information regarding genetic functions and evolution (Ran et al., 2010; Li et al., 2018; Eily et al., 2019). In line with this, DNA microarray technology can now be used for analysis of expressed genes of microbes from multiple genomes simultaneously using probes to determine up or downregulated genes. Alternatively, RNA sequencing (RNA-Seq) of cyanobiont genomes approximately 5.4–9.0 Mbp – in obligate cyanobiont *Nostoc azollae* and facultative cyanobiont *Nostoc punctiforme* PCC 73102 of *Azolla filiculoides*, respectively (Ran et al., 2010) and 6.7 Mbp in *Nostoc cycadae* of *Cycas revoluta* (Kanesaki et al., 2018) – can be utilized that could detect new genes and splicing events. Aside from ruling out the need for manual separation of microbe from host tissues, Next generation sequencing (NGS) technology provides high resolution data. Likewise, tissue-specific whole transcriptome profiling might be applied to coralloid root tissues with genome size of about 20–30 Gbp (Wang et al., 2018) for determining expressed genes during symbiosis. Furthermore, gaining bioinformatics skills is necessary to maximize the analysis of the outputs obtained from huge amounts of genomic data.

Cyanobacteria are also valuable sources of metabolites (Haque et al., 2017). Thus, venturing into cycad and/or cyanobiont metabolomics research might help to identify useful products, such biofertilizers, antimicrobials and other natural products of economic importance. It may also facilitate studies on cyanotoxins and biomagnification theories. Likewise, metabolites are also believed to play major roles in cell signaling and communication and could also assist in providing insights on symbiotic pathways and identification of enzymes involved in symbiosis. Many areas of cycad-cyanobacteria symbiosis are still waiting to be explored. With technology rapidly advancing simultaneously with the skills of researchers, restrictions previously deemed overwhelming currently appear to be promising.

## Author Contributions

TC and AC designed the study. AC gathered the materials and wrote the manuscript. TC, NL, and JD reviewed the manuscript. All authors read and approved the final manuscript.

## Conflict of Interest Statement

The authors declare that the research was conducted in the absence of any commercial or financial relationships that could be construed as a potential conflict of interest.
